# SOLiD sequencing of four *Vibrio vulnificus *genomes enables comparative genomic analysis and identification of candidate clade-specific virulence genes

**DOI:** 10.1186/1471-2164-11-512

**Published:** 2010-09-24

**Authors:** Paul A Gulig, Valérie de Crécy-Lagard, Anita C Wright, Brandon Walts, Marina Telonis-Scott, Lauren M McIntyre

**Affiliations:** 1 Department of Molecular Genetics and Microbiology, University of Florida, Gainesville, Florida, USA; 2 Department of Microbiology and Cell Science, University of Florida, Gainesville, Florida, USA; 3 Department of Food Science and Human Nutrition, University of Florida, Gainesville, Florida, USA; 4 Department of Genetics, University of Melbourne, 3010 Australia

## Abstract

**Background:**

*Vibrio vulnificus *is the leading cause of reported death from consumption of seafood in the United States. Despite several decades of research on molecular pathogenesis, much remains to be learned about the mechanisms of virulence of this opportunistic bacterial pathogen. The two complete and annotated genomic DNA sequences of *V. vulnificus *belong to strains of clade 2, which is the predominant clade among clinical strains. Clade 2 strains generally possess higher virulence potential in animal models of disease compared with clade 1, which predominates among environmental strains. SOLiD sequencing of four *V. vulnificus *strains representing different clades (1 and 2) and biotypes (1 and 2) was used for comparative genomic analysis.

**Results:**

Greater than 4,100,000 bases were sequenced of each strain, yielding approximately 100-fold coverage for each of the four genomes. Although the read lengths of SOLiD genomic sequencing were only 35 nt, we were able to make significant conclusions about the unique and shared sequences among the genomes, including identification of single nucleotide polymorphisms. Comparative analysis of the newly sequenced genomes to the existing reference genomes enabled the identification of 3,459 core *V. vulnificus *genes shared among all six strains and 80 clade 2-specific genes. We identified 523,161 SNPs among the six genomes.

**Conclusions:**

We were able to glean much information about the genomic content of each strain using next generation sequencing. Flp pili, GGDEF proteins, and genomic island XII were identified as possible virulence factors because of their presence in virulent sequenced strains. Genomic comparisons also point toward the involvement of sialic acid catabolism in pathogenesis.

## Background

*Vibrio vulnificus *is an opportunistic pathogen that causes sepsis in humans after ingestion of contaminated raw oysters or wound infection and necrotizing fasciitis from contamination of wounds (for a review see [1,2]). The mortality rates for sepsis and wound infection are ~50% and ~15%, respectively. During infection of humans the bacteria replicate rapidly, extensively invade tissues, and cause severe tissue destruction. Mouse models of infection coupled with molecular genetic analysis have identified several virulence factors partially explaining the high mortality and extreme tissue destruction, most importantly, polysaccharide capsule [3,4], RtxA1 toxin [5-7], acquisition of iron [8,9], pili [10,11], and flagella [12,13]. However, these factors do not completely explain the remarkable virulence of *V. vulnificus*.

*V. vulnificus *can be classified in several different manners. One of the first classification schemes was based on biochemical reactions of strains initially yielding two biotypes: biotype 1 most often associated with contamination of oysters and causing human disease and biotype 2 associated with infection of eels [14]. Recently, a third biotype that caused wound infection from handling fish in Israel was identified [15]. Genetic analysis using analysis of ribosomal RNA loci [16,17], multilocus sequence typing (MLST) [18-20], and virulence-correlated gene (*vcg*) PCR [21] revealed that *V. vulnificus *strains could be divided into two groups. While the descriptors for these two groups vary (clades, populations, clusters, and lineages), the terms clade 1 and clade 2 are used here to follow the MLST clusters of Bisharat et al. [19]. Biotype 1 strains are present in both clades, whereas biotype 2 strains are present only in clade 1. Based on MLST analysis, biotype 3 strains appear to be a hybrid between clades 1 and 2 [18]. Clade 1 strains are most often isolated from environmental samples, while clade 2 strains are most often associated with human disease. Because of these epidemiological patterns, many investigators hypothesized that clade 2 strains possess inherently greater virulence. In an analysis of 69 biotype 1 *V. vulnificus *strains, we recently determined that both clade 1 and clade 2 strains have the ability to cause severe skin infection in subcutaneously inoculated iron dextran-treated mice (Thiaville, P.C. et al., Infect. Immun, submitted; Jones, M. et al., in preparation). The major distinction between the clades was that clade 2 strains had a greater propensity to cause systemic infection and death in the mouse model, although there were some attenuated clade 2 strains and highly virulent clade 1 strains.

Analysis of the genomic DNA sequences of clade 1 and clade 2 strains would contribute to the identification of genetic differences among strains. As microbes engage in lateral gene transfer and are often highly divergent in genomic content, this study could help identify genes responsible for the differences in virulence between these clades. Both of the complete and annotated *V. vulnificus *genomes are of clade 2 strains, CMCP6 (GenBank accession numbers AE016795 and AE016796) and YJ016 (GenBank accession numbers BA000037, BA000037, AP005352). The lack of genomic sequence data from clade 1 strains is a serious impediment to understanding the differences in virulence between the two clades and in dissecting the virulence of *V. vulnificus *in general. We therefore undertook the present study to rapidly and economically obtain genomic sequence of numerous *V. vulnificus *strains representing both clades and the two major biotypes.

We hypothesized that clade 2 strains are more virulent, at least in part, because they contain unique virulence genes that are missing in most clade 1 strains. Therefore, identifying DNA sequences common to clade 2 strains and missing from clade 1 strains would create a set of putative virulence genes that could be subsequently experimentally examined. Because of the propensity of clade 1 strains to be associated with oysters, these strains may possess unique genes enabling colonization of shellfish. Therefore, unique clade 1 genes offer insight into the Vibrio-oyster relationship. However, not all genes uniquely associated with one clade will be involved with interactions with animal hosts, and virulence genes will not necessarily be present only in virulent genotypes. An example of the former is that the ability of *V. vulnificus *to ferment mannitol is associated with the cluster of strains that we are calling clade 2 most often derived from clinical cases [22], and an example of the latter is the nearly universal presence of the RtxA1 toxin in both virulent and attenuated *V. vulnificus *strains (Joseph, J.L., et al., in preparation). Finally, by comparing the genomes of a variety of strains representing the different clades and biotypes, the set of genes in the *V. vulnificus *genome shared by all *V. vulnificus *strains can be identified. Over and above identifying relationships between the presence and/or absence of genes among strains, identifying single nucleotide polymorphisms (SNPs) could also reveal the genetic basis for differential virulence and shellfish-colonizing phenotypes, as well as other phenotypes.

Given these goals, we used the SOLiD sequencing system on four *V. vulnificus *strains, each of which represented a unique genotype/virulence phenotype combination (Table 1). *V. vulnificus *M06-24/O [4] is a typical clade 2 strain exhibiting a high level of virulence in the subcutaneously inoculated iron dextran-treated mouse model [23-25]. Strain 99-520 DP-B8 [25] is a typical clade 1 strain that can infect skin tissues but is defective at causing systemic infection and death in the mouse model. Strain 99-738 DP-B5 [25] is an unusual clade 1 strain that is highly virulent in the mouse model, causing systemic infection and death. ATCC 33149 [26] is typical biotype 2 strain isolated from an eel. Using SOLiD sequencing enabled us to obtain approximately 100X coverage with 35-nt reads among four genomes. This selection of strains analyzed with SOLiD sequencing enabled comparative genomics to be performed and identified clade 2-specific genomic sequences and the genes of *V. vulnificus *shared among all of the strains sequenced to date.

**Table 1:** Genotypes and virulence phenotypes of the *V. vulnificus *strains whose genomes were sequenced in this study*.

Strain	Source	Biotype	MLST*	vcg*	*rrn*	rep-PCR*	Skin Infection	Liver Infection/Death
M06-24/O	Clinical	1	2	C	B	8	+	+
99-520 DP-B8	Oyster	1	1	E	AB	7	+	-
99-738 DP-B5	Oyster	1	1	E	A	7	+	+
ATCC 33149	Eel	2	1	E	A	5	-	-

*Virulence data for biotype 1 strains are from Thiaville, P.C., et al. (Infect. Immun., submitted) and for ATCC 33149 are from this study. MLST, *vcg*, and rep-PCR data are from Mahmud, et al. [83]. *rrn *data for biotype 1 strains are from Thiaville, P.C., et al. (Infect. Immun., submitted) and for ATCC 33149 are from Vickery et al. [84].

## Results

### Numbers of SOLiD sequencing reads

We performed SOLiD sequencing on the genomes of four *V. vulnificus *strains to increase the understanding of the genetic differences between the two major clades and the biotypes of this organism and to possibly identify sequences associated with differences in virulence potential in our subcutaneously inoculated iron dextran-treated mouse model [23-25]. *V. vulnificus *99-520 DP-B8 and 99-738 DP-B5 are clade 1 strains, typically isolated from environmental sources. Strain 99-520 DP-B8 exhibits the typical attenuated virulence of clade 1 strains, i.e., it can cause skin infection but is defective at causing systemic infection and death. In contrast, strain 99-738 DP-B5 exhibits a high level virulence more characteristic of clade 2 strains, i.e., it causes skin infection, systemic infection, and death (Thiaville, P.C., et al., Infect. Immun., submitted). *V. vulnificus *M06-24/O is a typical clade 2 strain with full virulence that has been widely used in examining molecular pathogenesis by many laboratories [4]. *V. vulnificus *ATCC 33149 is a biotype 2 strain isolated from an eel [26]. Genomic DNA from each of these strains was loaded onto one-fourth of a 25 mm × 75 mm SOLiD™ slide for sequencing on an Applied Biosystems SOLiD™ apparatus at the University of Florida Interdisciplinary Center for Biotechnology Research, as described in the Methods. The total numbers of 35-bp reads for each strain were as follows: 99-520 DP-B8 - 3.16 × 10^7^, 99-738 DP-B5 - 3.21 × 10^7 ^, M06-24/O - 3.50 × 10^7^, and ATCC 33149 - 3.38 × 10^7^. These totals represented putative 210- to 239-fold coverage of each of the genomes, on the assumption that all of the data were usable. The reads from each of the four newly sequenced genomes have been deposited into the NCBI Short Read Archive (accession number SRA009283.2).

### Comparison of SOLiD sequencing reads to reference *V. vulnificus *genomes

**Figure 1:**
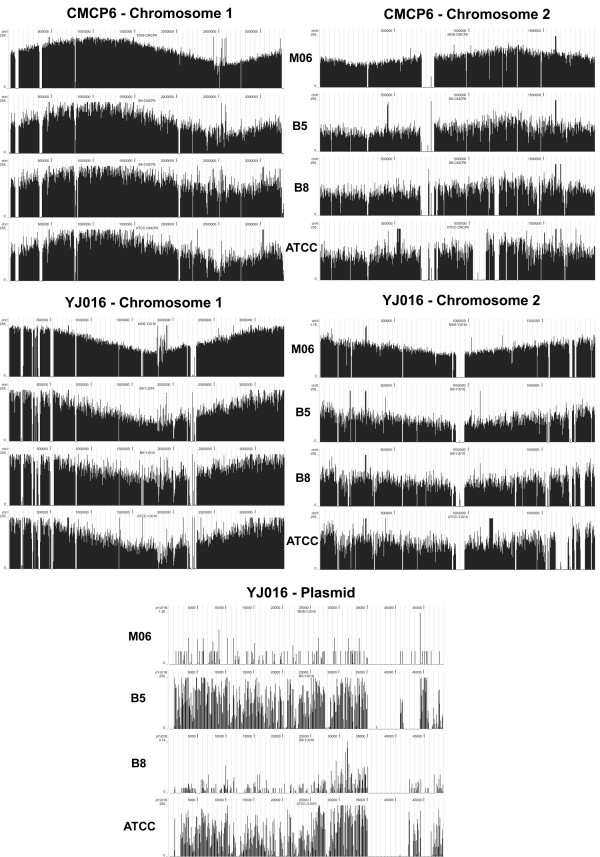
**Graphical representation of coverage of the reference genome components by sequences of each of the four newly sequenced genomes**. The depth of coverage (number of matched 35-nt reads per 100-nt window of the reference genomes) is plotted for both chromosomes of the reference CMCP6 and YJ016 genomes and the YJ016 plasmid. The source strain for the reads being matched are as follows: M06 - M06-24/O, B5 - 99-738 DP-B5, B8 - 99-520 DP-B8, ATCC - ATCC 33149. It should be noted that coverage of the reference genomes is not as continuous as it appears in the figures.

Reads were mapped onto the two reference *V. vulnificus *genomes, CMCP6 and YJ016 using MAQ [27]. This analysis enabled the identification of DNA sequences and ORFs that were present in the newly sequenced strains that have already been described in the reference strains. Graphical representation of the coverage of the CMCP6 and YJ016 genomes by the reads from each of the four newly sequenced *V. vulnificus *strains is shown in Figure 1.

We then mapped reads to plasmids described for biotype 2 *V. vulnificus *and whose DNA sequences are known (pR99, pC4602-1, and pC4602-2) [28]. As expected, greater than 90% of all three of these reference sequences were matched to reads from biotype 2 ATCC 33149, and lesser homologies were observed for the biotype 1 strains (Additional File 1, Table S1). For clade 1 strains, between 37 and 56% of these plasmid sequences matched with 99-738 DP-B5, and only 6 to 20% of plasmid sequenced matched the SOLiD reads from strain 99-520 DP-B8. These results suggested that 99-738 DP-B5 would have a plasmid, whereas 99-520 DP-B8 would not, and we confirmed this by gel electrophoresis of extracted plasmid DNA (data not shown). The reads from strain M06-24/O, which is a clade 2 strain and least related to the other strains, only matched to 1% of plasmid pC4602-1 and failed to match to any sequences of plasmids pC4602-2 and pR99. This is in agreement with M06-24/O not having a plasmid [29].

Despite the prediction of approximately 210-fold coverage based on the raw number of reads obtained for each genome, coverage was actually on the order of 100-fold. In total, 45% to 64% of the raw sequencing reads mapped to one of the two reference genomes, leaving a considerable number of unmapped reads. Some of these reads were of low complexity and may represent sequencing error. Because approximately 14% of both CMCP6 and YJ016 are low complexity, these unmapped reads also may be derived from regions of low complexity in the sequenced genomes. It is a limitation of the short read technology that we cannot distinguish among these scenarios. For the remaining unmapped reads that were not of low complexity, there are two possibilities: these reads represented truly unique sequences for the newly sequenced genomes or these reads were errors in the sequencing system. In an attempt to separate these two possibilities, these unmapped reads were compared to several bacterial genomes by mapping the reads in SOLiD colorspace using MAQ [27]. This would identify orthologs of *V. vulnificus *strains in other species. The largest number of matches (273,045) was found with the genomic sequence of *V. cholerae *NC16961 (GenBank accession numbers AE003852 and AE003852). (Additional File 2, Table S2). These *V. cholerae *matches yielded 20 genes in total from the four sequenced genomes. Of these *V. cholerae *genes, sixteen were identified from only a single *V. vulnificus *strain. Other novel genes may still be found, but they would be genes not previously identified in any other bacterial genomes.

There were between 15 and 22 million unmatched reads for each of the newly sequenced genomes. The cause of such a large amount of data with no similarity to known genes cannot be explained by low complexity alone, as many of these reads are not of low complexity. While it remains possible that novel genes are included in these data, it is also possible that these reads are just noise from the technology.

Figure 1, which graphically shows the coverage of the reference genome elements by each of the newly sequenced genomic reads, reveals large regions on each of the reference genomic elements for which there were no matched reads from each of the newly sequenced genomes. Detailed comparisons of coverage generated lists of the genes of CMCP6 and YJ016 lacking significant depth of coverage from the newly sequenced reads (Additional File 3, Table S3A and Additional File 4, Table S3B, respectively). There were 309 ORFs unique to CMCP6 and 489 ORFS unique to YJ016 relative to the other five sequenced strains. In CMCP6 chromosome 1, two large regions that were not present in any of the four newly sequenced genomes included genes VV1_0063 to VV1_0124 and VV1_0374 to VV1_0400. These regions, which were also missing from YJ016, appear to encode phage genomes. They contain genes annotated as bacteriophage phi 1.45 protein-like protein (VV1_0066), P2-like prophage tail protein × (VV1_0086), phage integrase (VV1_0372), or they resembled other mobile genetic elements with putative transposases (VV1_0385, VV1_0386).

Another CMCP6-specific region spanned the beginning and ends of chromosome 1 (genes VV1_0001 to VV1_0011 and VV1_3192 to VV1_3205). This region also appeared to encode a phage or other mobile genetic element. A smaller CMCP6-specific region located at genes VV1_0777 to VV1_0781 appeared to encode sugar metabolism genes possibly involved in lipopolysaccharide (LPS) or capsular biosynthesis. CMCP6 chromosome 2 contained a very large region at genes VV2_0630 to VV2_0712 not present in any other strains. This region appeared to have been derived from a mobile genetic element, either a phage or transposon. There were also smaller regions unique to CMCP6 on chromosome 2.

The YJ016 genome similarly contained numerous regions that were not present in any of the other newly sequenced genomes. On chromosome 1, YJ016-specific genes were located at VV0130 to VV0165, VV0343 to VV0367, VV0514 to VV0559, VV0799 to VV0817, and VV2191 to VV2262. The largest of these YJ106 regions at VV2191 to VV2262 appeared to be phage-related. A similar pattern was evident for YJ016 chromosome 2. A very large YJ106-specific region spanning VVA0825 to VVA0888 was notable. This region consisted mainly of hypothetical proteins, but there is a possibility that this region is phage-related, as VVA0886 is annotated as a phage integrase.

The coverage of the YJ106 plasmid, which encodes 69 genes, was very different among the four newly sequenced genomes. The genomes containing the most matches were 99-738 DP-B5 and ATCC 33149, with 50 and 44 genes, respectively, matched to the YJ016 plasmid. As expected, both 99-738 DP-B5 and ATCC 33149 contain plasmids. None of the YJ016 plasmid genes matched to the reads of 99-520 DP-B8 or M06-24/O, neither of which contains plasmids.

*V. vulnificus*, like other *Vibrio *species, encodes a super-integron on its large chromosome [30]. Integrons are specific regions of genomic sequence that have the ability to accumulate gene cassettes via site-specific recombination [31]. They are located in genomes at *attI *sites and contain a site-specific integrase, *intI*, that mediates acquisition of gene cassettes at repetitive *attC *sites, which are generally conserved among closely related bacteria. The vibrio integrons are called super-integrons because of their unusually large sizes [32]. In CMCP6 the super-integron spans genes VV1_2401 to VV1_2501, and in YJ016 the super-integron spans genes VV1745 to VV1941. As shown in Additional File 3, Table S3A and Additional File 4, Table S3B, the genes encoded within these super-integrons are mostly strain-specific, not having significant homology with the four newly sequenced genomes or each other. It is interesting that the super-integrons did not appear in Figure 1 as missing from the newly sequenced genomes, most likely because of the *attC *sites and presence of infrequent homologous genes between the genomes.

In contrast to identifying sequences missing from the newly sequenced genomes, we also identified the genes shared among all of the six genomes, thereby identifying the core *V. vulnificus *genome. Up to this point, shared genes based on the two reference genomes numbered 3,915 genes. Adding our four newly sequenced genomes, there are 3,459 genes common to all sequenced *V. vulnificus *strains, listed in Additional File 5, Table S4. The number of shared genes can only get smaller as more genomes are sequenced. Since there are 4,473 protein-coding genes in the CMCP6 genome and 5,024 protein-coding genes in the YJ016 genome, but only 3,915 genes shared between them, there is clearly an enormous amount of strain-specific sequence among these clade 2 strains. The frequency of hypothetical proteins in the core genome was 20.3% compared with the overall frequency of 23.6% in the CMCP6 genome.

The total number of genes obtained by combining the CMCP6 and YJ016 reference genomes and excluding redundancy is 5,630. Among the 4,473 genes in the CMCP6 genome, 309 (6.9%) were unique to this strain, and among the 5,026 genes in the YJ016 genome, 489 (9.7%) were unique to this strain relative to all of the other genomes. By combining the matches for each strain with the reference genomes we identified the following numbers of genes for each strain: ATCC 33149 - 4,184; 99-738 DP-B5 - 4,359; 99-520 DP-B8 - 4,225; and M06-24/O - 4,534.

### Genomic inference of different *V. vulnificus *genotypes

We asked which genes were common only to the three biotype 1/clade 2 strains, but not present in the two biotype 1/clade 1 strains or the biotype 2 strain, because this could help identify the genes that are responsible for the increased virulence of clade 2 strains (Thiaville, P.C. et al., Infect. Immun., submitted). The 80 clade 2-specific genes are listed in Table 2. Among the notable clade 2-specific genes and regions are several GGDEF proteins (genes VV1_2061, VV1_2228, VV1_2321 in the CMCP6 genome) and a Flp pilus-coding region (genes VV1_2330 to VV1_2337 in the CMCP6 genome). GGDEF proteins are involved with signal transduction in many bacteria by regulating intracellular levels of the signaling molecule cyclic-di-GMP [33], and Flp pili could be involved with adherence or genetic exchange [34]. Hypothetical proteins comprised 36.3% of the clade 2-specific genes, compared with the overall frequency of 23.6% of hypothetical proteins in the CMCP6 genome. Because the reference strains are both clade 2, any clade 1-specific genes will be missed in this initial mapping.

**Table 2:** Clade 2-specific genes

Tag	Product	Gene	Cog
VV1_0456	putative transcriptional regulator	-	COG0583K
VV1_0457	hypothetical protein VV1_0457	-	-
VV1_0458	hypothetical protein VV1_0458	-	-
VV1_0459	hypothetical protein VV1_0459	-	-
VV1_0465	exopolyphosphatase	-	COG0248FP
VV1_0515	hypothetical protein VV1_0515	-	COG3930S
VV1_0766	hypothetical protein VV1_0766	-	-
VV1_0789	hypothetical protein VV1_0789	-	-
VV1_1090	hypothetical protein VV1_1090	-	-
VV1_1094	chromosome segregation ATPase	-	-
VV1_1095	Serine/threonine protein kinase	-	COG0515RTKL
VV1_1518	3-methyladenine DNA glycosylase	-	COG0122L
VV1_1751	hypothetical protein VV1_1751	-	-
VV1_2031	Type I restriction enzyme EcoEI M protein	-	COG0286V
VV1_2037	Type I restriction enzyme EcoEI R protein	-	COG4096V
VV1_2038	transcriptional regulator	-	-
VV1_2061	GGDEF family protein OMPH_PHOPR porin-like protein H	-	COG2199T
VV1_2114	precursor	-	COG3203M
VV1_2115	hypothetical protein VV1_2115	-	COG3110S
VV1_2158	methyl-accepting chemotaxis protein	-	COG0840NT
VV1_2183	hypothetical protein VV1_2183	-	COG2378K
VV1_2184	ATPase involved in DNA repair	-	COG0419L
VV1_2228	GGDEF family protein	-	COG3706T
VV1_2321	GGDEF family protein	-	COG3614T
VV1_2326	hypothetical protein VV1_2326	-	-
VV1_2327	hypothetical protein VV1_2327	-	-
VV1_2329	hypothetical protein VV1_2329	-	-
VV1_2330	Flp pilus assembly protein CpaB	-	COG3745U
VV1_2331	Flp pilus assembly protein	-	COG4964U
VV1_2332	hypothetical protein VV1_2332	-	-
VV1_2333	pilus assembly protein CpaE-like protein	-	COG4963U
VV1_2334	Flp pilus assembly protein	-	COG4962U
VV1_2335	Flp pilus assembly protein TadB	-	COG4965U
VV1_2336	Flp pilus assembly protein TadC	-	COG4965U
VV1_2337	Flp pilus assembly protein TadD	-	COG5010U
VV1_2338	hypothetical protein VV1_2338	-	-
VV1_2339	hypothetical protein VV1_2339	-	COG4961U
VV1_2340	hypothetical protein VV1_2340	-	-
VV1_2341	azoreductase	acpD	COG1182I
VV1_2401	super-integron integrase IntIA	-	COG4974L
VV1_2708	hypothetical protein VV1_2708	-	-
VV1_2748	response regulator	-	COG3437KT
VV1_2758	amino acid transporter	-	-
VV1_2840	NhaP-type Na+/H+ and K+/H+ antiporters	-	COG0025P
VV1_2868	methyl-accepting chemotaxis protein	-	COG0840NT
VV1_3144	hypothetical protein VV1_3144	-	-
VV2_0019	alcohol dehydrogenase	-	COG1454C
VV2_0073	anti-anti-sigma regulatory factor	-	COG1366T
VV2_0074	anti-anti-sigma regulatory factor	-	COG1366T
VV2_0075	anti-sigma regulatory factor	-	COG2172T
VV2_0076	Serine phosphatase RsbU	-	-
VV2_0077	FOG: CheY-like receiver	-	COG0642T
VV2_0078	response regulator AraC-type DNA-binding domain-containing	-	COG3437KT
VV2_0212	protein	-	COG2207K
VV2_0312	hypothetical protein VV2_0312	-	-
VV2_0313	response regulator	-	COG0745TK
VV2_0627	hypothetical protein VV2_0627 AraC-type DNA-binding domain-containing	-	COG2378K
VV2_0782	protein	-	COG2207K
VV2_0783	major facilitator superfamily permease	-	-
VV2_0851	hypothetical protein VV2_0851	-	COG0845M
VV2_0864	hypothetical protein VV2_0864	-	-
VV2_0868	acetyltransferase	-	COG0456R
VV2_0881	long-chain fatty acid ABC transporter	-	COG2067I
VV2_0884	Mg2+ and Co2+ transporter	-	-
VV2_0993	transcriptional regulator	-	COG0583K
VV2_0994	multidrug resistance efflux pump	-	COG1566V
VV2_1075	dehydrogenase	-	COG1028IQR
VV2_1138	hypothetical protein VV2_1138	-	COG3904S
VV2_1149	hypothetical protein VV2_1149	-	-
VV2_1186	transcriptional regulator	-	COG0583K
VV2_1203	hypothetical protein VV2_1203	-	COG3930S
VV2_1204	glutathione synthetase	-	COG0189HJ
VV2_1273	transcriptional regulator	-	COG0583K
VV2_1274	Ca2+/H+ antiporter	-	COG0387P
VV2_1275	hypothetical protein VV2_1275	-	COG0586S
VV2_1290	hypothetical protein VV2_1290	-	COG0834ET
VV2_1303	hypothetical protein VV2_1303	-	-
VV2_1304	Beta-glucosidase-related glycosidase	-	COG1472G
VV2_1309	DMT family permease	-	-
VV2_1363	transcriptional regulator	-	COG0583K

Strain 99-520 DP-B8 is a typical clade 1 strain with attenuated virulence, while strain 99-738 DP-B5 is a clade 1 strain with high virulence typical of clade 2 strains. There were 61 genes in 99-738 DP-B5 that were common to the three clade 2 strains but missing from attenuated clade 1 strain 99-520 DP-B8 and biotype 2 strain ATCC 33149. (Table 3). This set of genes could contain virulence genes acquired by 99-738 DP-B5 that endow it with clade 2-like virulence. Hypothetical proteins comprised 19.7% of this set of genes, compared with the overall frequency of 23.6% hypothetical proteins in the CMCP6 genome. It is noteworthy that the clade 2 + 99-738 DP-B5 specific set of genes includes genomic island XII identified by Cohen et al. [20] as being present in most clade 2 strains and missing from most clade 1 strains (genes VV2_1090 to VV2_1111 on the CMCP6 genome). They hypothesized that genomic island XII could be responsible for the putative differential virulence of clade 2 strains, evidenced by their association with clinical cases.

**Table 3:** Genes common to *V. vulnificus *99 738 DP B5 and clade 2 strains

Tag	Product	Gene	cog
VV1_0251	hypothetical protein VV1_0251	-	COG3094S
VV1_0411	choline-glycine betaine transporter	-	COG1292M
VV1_0638	mannitol/fructose-specific phosphotransferase system IIA protein	-	COG2213G
VV1_0639	mannitol-1-phosphate 5-dehydrogenase	-	COG0246G
VV1_0640	mannitol repressor protein	mtlR	COG3722K
VV1_0641	D-fructose-6-phosphate amidotransferase	-	COG0449M
VV1_0834	DMT family permease	-	-
VV1_0835	hypothetical protein VV1_0835	-	-
VV1_1655	H+/gluconate symporter	-	COG2610GE
VV1_1656	sugar diacid utilization regulator	-	COG3835KT
VV1_2188	helicase-related protein	-	COG1061KL
VV1_2189	tellurite resistance protein-related protein	-	COG2227H
VV1_2744	response regulator	-	COG2197TK
VV1_2936	putative transcriptional regulator	-	-
VV2_0151	arylsulfatase A	-	COG3119P
VV2_0335	methyl-accepting chemotaxis protein	-	COG0840NT
VV2_0542	manganese transporter NRAMP	-	COG1914P
VV2_0726	hypothetical protein VV2_0726	-	COG3055S
VV2_0729	transcriptional regulator	-	COG1737K
VV2_0730	dihydrodipicolinate synthase/Nacetylneuraminate lyase	-	COG0329EM
VV2_0731	TRAP-type C4-dicarboxylate transport System	-	COG1593G
VV2_0732	TRAP-type C4-dicarboxylate transport system	-	COG3090G
VV2_0733	TRAP-type C4-dicarboxylate transport system	-	COG1638G
VV2_0734	N-acetylmannosamine-6-phosphate 2- epimerase	-	COG3010G
VV2_0735	N-acetylmannosamine kinase	-	COG1940KG
VV2_0736	N-acetylglucosamine-6-phosphate deacetylase	-	COG1820G
VV2_0892	diadenosine tetraphosphate hydrolase	-	COG0537FGR
VV2_0893	arsenite efflux pump ACR3	-	COG0798P
VV2_0894	transcriptional regulator	-	COG0640K
VV2_0920	amidohydrolase	-	COG0388R
VV2_0988	hypothetical protein VV2_0988	-	-
VV2_0989	Alkyl sulfatase	-	COG2015Q
VV2_1035	ABC transporter permease	-	COG3932R
VV2_1090	hypothetical protein VV2_1090	-	-
VV2_1091	hypothetical protein VV2_1091	-	-
VV2_1092	hypothetical protein VV2_1092	-	-
VV2_1093	2-deoxy-D-gluconate 3-dehydrogenase	-	COG1028IQR
VV2_1094	galactose-1-phosphate uridylyltransferase	-	COG1085C
VV2_1095	UDP-glucose 4-epimerase	-	COG1087M
VV2_1096	Sulfate permease	-	COG0659P
VV2_1097	hypothetical protein VV2_1097	-	-
VV2_1098	CBS domain-containing protein	-	COG3448T
VV2_1099	methyl-accepting chemotaxis protein	-	COG0840NT
VV2_1100	ATPase component of various ABC-type transport system	-	COG1123R
VV2_1101	ABC-type dipeptide/oligopeptide/nickel transport system	-	COG1173EP
VV2_1102	ABC-type dipeptide/oligopeptide/nickel transport system	-	COG0601EP
VV2_1104	ABC-type dipeptide transport system	-	COG0747E
VV2_1105	hypothetical protein VV2_1105	-	COG4289S
VV2_1106	arylsulfatase A	-	COG3119P
VV2_1107	arylsulfatase regulator	-	COG0641R
VV2_1108	arylsulfatase A	-	COG3119P
VV2_1109	arylsulfatase A	-	COG3119P
VV2_1110	hypothetical protein VV2_1110	-	-
VV2_1259	hypothetical protein VV2_1259	-	-
VV2_1403	GGDEF domain-containing protein	-	COG2199T
VV2_1505	hypothetical protein VV2_1505	-	COG1233Q
VV2_1508	putative two-component response regulator	-	COG2197TK
VV2_1509	GGDEF family protein	-	COG2199T
VV2_1510	response regulator	-	COG2197TK
VV2_1511	response regulator VieA	-	COG2200T
VV2_1512	sensor kinase VieS	-	COG0642T

Within genomic island XII are paralogs of galactose utilization genes (VV2_1095, a paralog of *galE2 *encoding UDP-glucose 4-epimerase and VV2_1094, a paralog of *galT2 *encoding galactose-1-phosphate uridylyltransferase) that are in an operon with a predicted sulfate transporter (VV2_1096). The canonical *galE *(VV1_1770) and *galT *(VV1_1771) are located elsewhere in the *galETKM *operon (VV_1770 to VV1_1773). The presence of additional *galET *genes in a subset of *V. vulnificus *strains with high virulence suggests a role for these genes in another metabolic pathway possibly benefitting the bacteria during infection.

The link with sulfate metabolism was intriguing because five other genomic island XII genes are annotated as arylsulfatase A (VV2_1106, VV2_1108, VV2_1109, and VV2_0151) or alkyl sulfatase (VV2_0989). These enzymes hydrolyze the sulfate from sulfated gangliosides (sulfatides). VV2_1098 and VV2_1110 in the genomic island encode chondroitinases (although they are not annotated as such in the reference genome sites). Sulfatides are important components of connective tissue involved with cell adhesion [35] and serve as the receptors for various microbial pathogens ranging from HIV [36], *Bordetella pertussis *[37], and *Helicobacter pylori *[38]. An arylsulfatase of *E. coli *K1 is necessary for invasion of the blood-brain barrier [39]; hence, such activity in virulent *V. vulnificus *strains could enable invasion through tissues, which is characteristic of *V. vulnificus *infection. In clinical *V. vulnificus *isolates, the presence of region XII, encoding arylsulfatases, chondroitinases, sulfate transport, and sulfate metabolism functions, suggests that this region may have an important scavenging function removing sulfate groups from host components, thereby providing sulfur and/or carbon sources, which could facilitate survival in the human host where free sulfur is limited. However, as noted above, some of the degradative enzymes in genomic island XII could also be involved in invasion of tissues. Cohen et al. [20] had noted the presence of such genes in genomic island XII predominant in the clade of *V. vulnificus *strains most associated with clinical strains. The exclusive presence of all of these genes in clade 2 plus the highly virulent clade 1 strain 99-738 DP-B5 suggests a role in virulence. The dissection of the roles in virulence, if any, played by these genomic island XII genes identified through our comparative genomic analysis will await construction and analysis of specific mutants. However, Bryant et al. [40] described the use of sodium dodecyl sulfate-polymyxin B-sucrose plates for the identification of *V. vulnificus *from shellfish samples. The ability of bacteria to form halos around colonies on this medium is indicative of alkyl sulfatase activity. In contrast to our determination that VV2_0989 is absent in the biotype 2 strain ATCC 33149 and clade 1 strain 99-520 DP-B8 and the results of Cohen et al. [20] similarly describing the limited presence of genomic island 12 among *V. vulnificus *strains, Bryant et al. observed that all 20 *V. vulnificus *strains examined possessed alkyl sulfatase activity. However, VV2_0885 and VV2_1032 are also annotated as alkyl sulfatase. Our results show that VV2_0885 is present in all six strains except 99-738 DP-B5 and VV2_1032 is present in all six strains. Hence, it would be expected that all *V. vulnificus *strains would exhibit alkyl sulfatase activity, in agreement with Bryant et al. [40].

Also of note in the clade 2 plus 99-738 DP-B5-specific genes not present in genomic island XII were linked genes possibly involved with sialic acid catabolism: N-acetylneuraminate lyase (NanA, VV2_0730), a TRAP transport system possibly involved with sialic acid transport (VV2_0731 to VV2_0733), N-acetylmannosamine-6-phosphate 2-epimerase (NanE, VV2_0734), N-acetylmannosamine kinase (NanK, VV2_0735), and N-acetylglucosamine-6-phosphate deacetylase (NagA, VV2_0736). Because the *nagB *gene (VV2_1200, glucosamine-6-P deaminase) is in the *V. vulnificus *core genome, the clade 2 strains and 99-738 DP-B5 uniquely have the ability to assimilate exogenous sialic acid into central metabolism as fructose-6-phosphate, relative to the other clade 1 strains and biotype 2 strains. However, *V. vulnificus *does not encode a neuraminidase (NanH) which would liberate sialic acid from host components. Almagro-Moreno and Boyd [41] had noted that sialic acid metabolism was unique to bacteria that interacted with mammalian hosts, either as pathogens or as commensals. Jeong et al. [42] recently constructed a *nanA *deletion in *V. vulnificus *and confirmed that the ability to utilize exogenous sialic acid was essential for virulence in intraperitoneally inoculated iron dextran-treated mice, as well as cytotoxicity in cell culture assays. They focused analysis of *nanA *on a single *V. vulnificus *strain and did not perform comparative genetics among strains of different genotypes or virulence phenotypes. The summation of these data regarding *nanA *is that our comparative genomic sequencing correctly identified unique virulence genes among different sets of *V. vulnificus*.

Another carbon source utilization pathway specific to the clade 2 plus 99-738 DP-B5 strains but not in genomic island XII is a complete mannitol catabolic pathway encoding the mannitol/fructose-specific phosphotransferase system IIA protein (*mtlABC*, VV1_0638), mannitol-1-phosphate 5-dehydrogenase (*mtlD*, VV1_0639), and a specific mannitol repressor (*mtlR*, VV1_0640). The significance of these genes to virulence is unknown. Interestingly, by examining 465 *V. vulnificus *strains, Drake et al. [22] previously determined that the ability to ferment mannitol by *V. vulnificus *was highly correlated with a strain being in, what we are calling, clade 2. Tison et al. [14] reported that biotype 2 strains were mannitol-negative. Our sequencing data, albeit on a considerably smaller sample size of strains, therefore corroborate the phenotypic analyses of these two previous studies.

### SNP analysis

In addition to the presence or absence of whole genes or blocks of genes, detailed above, genetic variation among the sequenced strains also consisted of nucleotide polymorphisms. We used MAQ to identify SNPs present in the newly sequenced genomes relative to the reference genomes. The SNPs from each of the pairwise analyses versus the reference genomes are listed in Additional Files 6, 7, 8, 9, 10, 11, 12, and 13, and the summary of the numbers of SNPs from each sequenced strain relative to the reference genomes is shown in Table 4. In examining SNPs, we did not exclude any sets of genes, such as putative mobile genetic elements, e.g., phages. It is interesting that M06-24/O, which had the highest amount of coverage relative to the reference genomes, had the lowest number of SNPs (mean of 42,191 SNPs per reference genome) compared with the other three strains (mean of 73,130 SNPs per reference genome). This likely reflects the fact that M06-24/O is in the same clade as the reference genomes.

**Table 4:** Numbers of SNPs from each of the four sequenced genomes relative to the two chromosomes of the reference genomes.

	CMCP6			YJ016		
		
	Chrom. 1	Chrom. 2	Total	Chrom. 1	Chrom. 2	Total
M06-24/O	23,752	17,390	41,142	25,530	17,709	43,239
99-738 DP-B5	46,469	27,457	73,926	46,833	27,152	73,985
99-520 DP-B8	46,059	26,440	72,499	46,156	26,223	72,379
ATCC 33149	46,259	26,355	72,614	47,549	25,828	73,377

The accuracy of the SOLiD-based SNPs in identifying polymorphisms was verified by examining Sanger sequencing of specific genomic regions of each of these strains. Having examined 8.7 kb of Sanger-derived sequence that contained SNPs identified from our SOLiD sequencing, we determined that 126 of 128 SNPs were accurately identified (98.4% accuracy).

**Figure 2:**
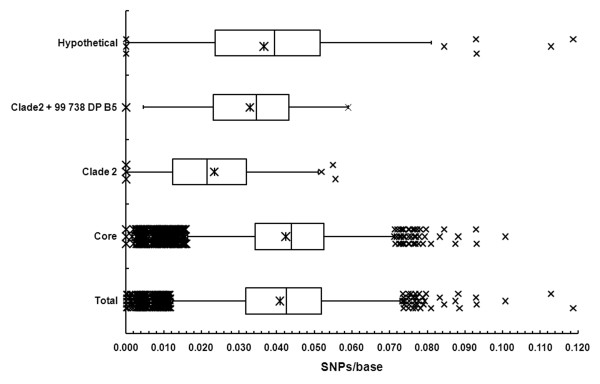
**Distribution of SNPs Relative to the CMCP6 Genome and Subsets of Genes**. Box and whisker plots of the SNPs/base for each of the subsets of annotated genes are shown.

We then examined the distribution of nonredundant SNPs among different sets of annotated ORFs using the CMCP6 reference genome. It must be emphasized that the vast majority of annotated ORFs have not been experimentally verified; hence, such an analysis is conjectural. Of the 201,981 nonredundant SNPs in the CMCP6 genome from all four sequenced strains, 177,464 fell within annotated ORFs (87.9%). This was not unexpected since this figure approximates the amount of the genome contained within annotated ORFs [30]. However, other interesting trends were evident. There were highly significant differences in the frequencies of SNPs between chromosomes 1 and 2 of CMCP6. Among the annotated ORFs, there were 0.037 SNPs/base for chromosome 1 and 0.044 SNPs/base in chromosome 2. Among the other sets of ORFs, there were significantly more SNPs/base in the core genome (0.043 SNPs/base) than in the total ORFs (0.040 SNPs/base) (Figure 2). As opposed to the inference that the core genome is actually more variable among strains, this difference most likely is due to the fact that the core genome, by definition, was shared among all of the sequenced strains; hence, had more shared sequences in which SNPs could be identified. In contrast, the lowest rate of SNPs was among the clade 2-specific genes, with only 0.019 SNPs/base. In the opposite manner to the core genome, this result would be expected since the clade 2-specific genes are unique and shared among the set of three genetically related clade 2 strains and because only one newly sequenced clade 2 strain, M06-24/O, contributed to this particular SNP pool. The frequency of SNPs in the clade 2 + 99-738 DP-B5 set of ORFs was 0.033 SNPs/base. The frequency of SNPs among hypothetical proteins (0.037 SNPs/base) was significantly lower than that of the total ORFs.

### Lineage-specific Expansions

Gu et al.[43]. recently reported an analysis of numerous *Vibrio *spp. to identify lineage-specific expansions (LSEs), genes that have been duplicated within a species or genotype. Some LSEs are specific to single strain, while others are present among varied strains across species. We examined some of the LSEs present in the reference genomes of *V. vulnificus *to determine if these loci are similarly present in the newly sequenced *V. vulnificus *genomes. We did not find a pattern to the presence or absence of the LSEs examined. For example, VV1_3196 and VV2_0703 form a pair of LSE genes in CMCP6. Neither of these genes has a homologue in YJ016 or any of the newly sequenced *V. vulnificus *genomes. In contrast, VV1_2851 and VV2_0347 constitute a pair of LSEs in CMCP6 that have homologues in YJ106 (VV1419 and VVA0904). The VV1_2851/VV1419 pair of genes has homologues in all of the four newly sequenced genomes, while VV2_0347 and VVA0904 do not.

## Discussion

This study is one of the first to use Applied Biosystems SOLiD sequencing for genomic sequencing of bacteria. Whole genome analysis has progressed considerably since the publication of the first complete DNA sequence of the pathogenic bacterium *Haemophilus influenzae *[44]. Until recently, the wealth of complete genomes available in public databases was decoded via the large-scale industrialization of the Sanger dideoxy chain-termination sequencing method [45,46]. The prospect of quickly and inexpensively resequencing large segments of the human genome or whole genomes of populations or species is driving development of a new generation of sequencing technologies with impacts in microbiology, functional genomics, ecology and evolutionary biology, human health, and beyond [45,47-51]. In particular, bacterial sequencing has been advanced by the high throughput, parallel format of the 454 Sequencer [51], the first 'next-generation' technology to *de novo *sequence and assemble whole bacterial genomes including *Mycoplasma genitalium *in a single machine run [52]. Bacterial comparative genomics has expanded rapidly owing to the speed of the 454 Sequencer compared to Sanger sequencing [53] as well as from a combination of the two technologies [54], while assessment of microbial diversity from complex communities (metagenomics) [55] has revealed insights into complex interactions such as mammalian obesity and the microbiome [56,57], the ocean biosphere [58], and the role of microbes in colony collapse disorder in honeybees [59].

More recently released 'second-generation' sequencing technologies such the Illumina GA2X Genome Analyzer (GA) and ABI SOLiD system have been developed [51]. To date, these next generation sequencing technologies generate shorter read lengths than Sanger sequencing, which poses a difficulty for *de novo *sequence assembly and defining large chromosomal rearrangements [49,51]. So far in prokaryotes, high quality draft sequences have been assembled in the absence of Sanger sequencing by combing the 454 and GA technologies [60-64]. Studholme et al. [65] utilized the Illumina platform alone for the *de novo *assembly of the draft genome sequence of *Pseudomonas syringae *pathovar *tabaci *strain 11528, revealing insights into the nature of type III protein-mediated pathogenicity.

The improved throughput from the massively parallel format of the new platforms (billions of bases in a single run) is ideal for revealing patterns of genetic variation among individuals by resequencing. For example, Srivatsan et al. [66] employed Illumina sequencing to improve the existing draft of the extensively studied model bacterium *Bacillus subtilis*, while also identifying polymorphisms between other well studied laboratory strains and their isolates. Moreover, this method was sensitive enough to identify typically difficult to isolate suppressor mutations in a single strain [66]. Using the same platform, whole-genome analysis of 12 isolates of the monomorphic human pathogen *Salmonella enterica *serovar Typhi revealed evolutionary loss of gene function consistent with the effects of genetic drift on a small effective population size [67]. Resequencing of the *Caernohabditis elegans *N2 Bristol strain and SNP discovery in another strain demonstrated the effectiveness of this technology in eukaryotes [68], and single base mutations in a mutant *C. elegans *strain were mapped, avoiding traditional genetic mapping efforts [69].

As one of the newer second-generation sequencers currently available, (although 'third-generation' single molecule sequencers are set to be marketed in 2010), the ABI SOLiD platform has been used more with eukaryotes than prokaryotes. One of the first studies focused on assessing cross-platform performance for sequence detection of known mutations in *C. elegans*. Comparable accuracy between GA and SOLiD for mapping the same *C. elegans *mutant strain as Sarin et al. [69] was reported [70]. Similarly, comparable accuracy was reported between 454, GA, and SOLiD methods for comparing a mutant strain of yeast to a reference genome [71]. At present the utility of the SOLiD platform is reflected in several resequencing studies in humans, including haplotype analysis, breakpoint mapping in disease-associated chromosomal rearrangements, and polymorphism discovery in protein coding exons [72-74]. With bacteria, SOLiD sequencing has been limited to verifying an *E. coli *reference strain sequence in conjunction with traditional sequencing [75], as well as resequencing of *Bacillus anthracis *strains for rapid and accurate forensic typing [76]. In our presently described study, the SOLiD platform was successfully utilized for rapid comparative genomic analysis of clade-specific and core genome sequences of the opportunistic pathogen *V. vulnificus*.

By examining the genomic DNA of each of four *V. vulnificus *strains on one-fourth of a SOLiD slide, we obtained 3.16 × 10^7 ^to 3.50 × 10^7 ^35-nt reads. This level of sequencing yielded approximately 100-fold coverage of each genome. Although the total numbers of reads would have predicted over 200-fold coverage, there was a significant amount of low complexity reads, as well as reads that were unmappable to the reference genomes.

We identified sequences that are unique to the highly virulent clade 2 strains. These 80 genes represent the set that could contain virulence genes that are responsible for the ability of clade 2 strains to cause systemic infection and death in subcutaneously inoculated iron dextran-treated mice (Thiaville, P.C., et al., Infect. Immun. submitted). Furthermore, we identified 61 additional genes that are common to the clade 2 strains and an unusual highly virulent clade 1 strain but absent from a typical attenuated clade 1 strain and a biotype 2 eel isolate. These 61 genes represent a very interesting set that could contain generally clade 2-specific genes that were acquired by a clade 1 strain and increased its virulence to that of typical clade 2 strains. Among these putative virulence genes were genomic island XII identified by Cohen et al. [20], and most interesting was a set of genes involved with sialic acid catabolism. Jeong et al. [42] recently determined that the ability to utilize sialic acid for metabolism was essential for virulence of *V. vulnificus*. We are currently examining the possible roles of several of these loci in virulence.

At the time of our performing this genomic sequence analysis, we had not performed virulence studies of biotype 2 ATCC 33149 in our subcutaneously inoculated iron dextran-treated mouse model for infection. However, Amaro et al. [77] previously reported that ATCC 33149 was of modest virulence in a different mouse model involving intraperitoneal infection. Based on our results indicating that ATCC 33149 lacked the genes shared among virulent clade 2 strains or clade 2 strains plus virulent clade 1 99-738 DP-B5, we hypothesized that ATCC 33149 would be attenuated for virulence in our model. In fact, when administered at the standard lethal dose of 1,000 CFU for virulent strains, ATCC 33149 caused only minimally detectable skin infection in one of five mice. Furthermore, when administered at 100 times the typical lethal dose (10^5 ^CFU/mouse), skin infection but no systemic infection or death ensued. Therefore, our genomic analysis of ATCC 33149 correctly predicted its attenuated virulence. It should be noted that Amaro and Biosca [78] reported that some biotype 2 strains are virulent for mammals, so the attenuation of ATCC 33149 was not a foregone conclusion.

Because phenotypic differences are not only rooted in presence or absence of whole genes, but also nucleotide polymorphisms, we generated a set of SNPs among the shared sequences of the reference and newly sequenced genomes (Table 4 and Additional Files 6, 7, 8, 9, 10, 11, 12, and 13). By examining Sanger-derived sequences for a subset of SNPs, we determined that 98.4% of our reported SNPs are accurate. Of the 128 SNPs examined, only two in one gene of one strain were not confirmed by Sanger sequencing.

Although the sample size of newly sequenced strains was small and each strain is a single representative of a unique genotype/virulence phenotype combination, some interesting relationships in SNPs were observed. Most interesting, the rate of SNPs was significantly higher for genes encoded on chromosome 2 compared with chromosome 1. Given that chromosome 1 of Vibrio is believed to encode most of the essential genes and that chromosome 2 is believed to have been acquired exogenously [79], it is reasonable that the highest rate of polymorphisms would occur in the chromosome 2. The number of SNPs between M06-24/O and the reference genomes was much lower than those from the other three genomes (Table 4), even though there were slightly more genes identified in M06-24/O. Because M06-24/O is in the same clade as the reference genomes, this result would be expected. Significant differences were observed in the frequencies of SNPs among about every subset of genes examined, e.g., clade 2-specific, core genome, hypothetical proteins (Figure 2). However, it must be noted that the numbers of strains contributing to the SNP pool for these subsets of genes differ between the sets. For example, the core genome is shared among all six strains, so all four newly sequenced strains contributed SNPs and could have generated a higher frequency of SNPs. In contrast, for the clade 2-specific genes, the only newly sequenced strain contributing SNPs, by definition of the subset, was M06-24/O.

By comparing the sequences shared among all six genomes, we identified the core *V. vulnificus *genome consisting of 3,459 genes. Gu et al. [43] examined the genomic sequences of all *Vibrio *species as of 2008 and identified 1,882 genes common to the genus. We are presently examining the core *V. vulnificus *genome to deduce possible metabolic and virulence characteristics of the species. We identified 20 genes previously unreported in *V. vulnificus *by using MAQ to compare the unmapped reads to the *V. cholerae *N16961 genome. If the clade 1 or biotype 2 genomes possessed sequences with sufficient similarity to the *V. cholerae *genome, we should have been able to identify and assemble them exactly as we did for the *V. vulnificus *reference genomes.

Most recently, Chun et al. [80] examined the genomes of 23 *V. cholerae *strains collected over 98 years. Their newly sequences genomes were obtained using a combination of Sanger and 454 sequencing. Like us, they based their phylogenetic relationships primarily on presence or absence of ORFs. Their analysis enabled the division of that species into 12 lineages, with one comprising the O1 strains and the seventh pandemic comprising a nearly identical clade. They determined that horizontal gene transfer significantly contributed to the evolution of the species.

## Conclusions

SOLiD sequencing of multiple bacterial genomes of *V. vulnificus *and subsequent comparative genomic analysis identified numerous genes that are common to the most virulent strains yet lacking from attenuated strains for which genomic DNA sequence data are available. These candidate virulence genes encode Flp pili, GGDEF proteins, and genomic island XII. Sialic acid catabolism was similarly identified as a potential contributory factor in molecular pathogenesis. These intriguing results will likely lead to more thorough understanding of molecular pathogenesis of *V. vulnificus*.

## Methods

### *V. vulnificus *strains

Each of the four *V. vulnificus *strains used for genomic sequencing was chosen to represent a specific combination of genotype and virulence phenotype. M06-24/O is a typical biotype 1, clade 2 strain that is highly virulent in our subcutaneously inoculated, iron dextran-treated mouse model. 99-520 DP-B8 is a typical biotype 1, clade 1 strain that is attenuated in our mouse model in that it can cause skin infection, but not systemic infection or death. 99-738 DP-B5 is an unusual biotype 1, clade 1 strain in that it is fully virulent in our mouse model. ATCC 33149 is a biotype 2 strain that is highly attenuated for virulence in our mouse model. These data are summarized in Table 1.

### SOLiD DNA sequencing

Sequencing runs were done using cycled ligation sequencing on a SOLiD™ Analyzer (Applied Biosystems, Beverly, MA) at the Interdisciplinary Center for Biotechnology Research at the University of Florida. Approximately 3 to 5 μg of purified bacterial genomic DNA was sheared into 80 to 100-bp short fragments with the Covaris™ S2 system according to the AB protocol. The sheared DNA was purified using a Qiagen MiniElute^® ^reaction cleanup kit. The purified sheared fragments were made blunt-ended with the Epicenter^® ^End-It™ DNA end-repair kit and subsequently ligated to short SOLiD P1 and P2 adapters (P1, 41-bp: 5'-CCACTACGCCTCCGCTTTCCTCTCTATGGGCAGTCGGTGAT-3'; P2, 23-bp: 5'-AGAGAATGAGGAACCCGGGGCAG-3'), which provide the primary sequences for both amplification and sequencing of the sample library fragments. Adapter-ligated DNA was then purified using the Agencourt kit. The reaction conditions were optimized to selectively bind DNA 100-bp and larger. At this point, DNA was nick-translated and resolved on 4% agarose gel, from which the 120 to 180-bp fragments were excised. The fractionated DNA was subjected to 8 to 10 cycles of PCR amplification. The number of PCR cycles needed for amplification was determined by the ability to visualize the amplified product on a 2.2% Lonza flash gel. The amplified PCR products were purified and then quantified using an Agilent 2100 bioanalyzer.

In preparation for sequencing, the DNA fragments were clonally amplified by emulsion PCR by using 1.6 billion, 1 μM beads with P1 primer covalently attached to the surface. Emulsions were broken with butanol, and ePCR beads were enriched for template-positive beads by hybridization with P2-coated capture beads (SOLiD reagent, Applied Biosystems). Template-enriched beads were extended at the 3' end in the presence of terminal transferase and 3' bead linker. About 60 million beads with clonally amplified DNA were then deposited onto one-fourth of a derivatized glass surface of a 25 mm × 75 mm SOLiD™ slide. The slide was then loaded onto a SOLiD instrument, and the 35-base sequences were obtained according to manufacturer's protocol.

### DNA sequence data management

The colorspace reads from SOLiD sequencing were aligned to the genomes of *V. vulnificus *strains CMCP6 (GenBank accession numbers AE016795 and AE016796) and YJ016 (GenBank accession numbers BA000037, BA000037, AP005352) using MAQ [27]. Reads from each of the four sample strains were mapped to the two reference sequences separately. Reads unmapped in both reference genomes were identified. Mapped reads were used to develop a consensus sequence for each of the four strains. For each strain relative to the two reference sequences, a gene was determined to be absent when the average depth of coverage over the open reading frame was less than 5X. Consensus sequences were also used to generate a list of SNPs among the six strains of *V. vulnificus *using the MAQ cns2snp [27].

Reads with low-complexity characteristics, defined as containing a homopolymer run of at least 5 bases, at least four repeats of the same dinucleotide in a row, or at least four repeats of the same trinucleotide in a row, were removed from the data set before further analysis. While these reads may represent true genomic regions, the difficulty in assigning them to a particular genomic region limits their value. This is an inherent problem with low complexity genomes and short read data. Reads unmapped in both reference sequences were then compared to the *V. cholerae *NC16961 reference genome using MAQ [27]. Windows of 100 nucleotides in the *V. cholerae *genome with a read depth of five or more were identified. Regions where five or more windows occurred in tandem were retained, while those with coverage less than five were discarded. Reads that initially mapped to a lower density area of the *V. cholerae *genome were re-examined for possible matches to the tandem windows.

In parallel to the *V. cholerae *exploration, the unmapped reads were examined for similarity to *V. vulnificus *biotype 2 plasmids pR99 (accession # AM293858), pC4602-1 (accession # AM293859), and pC4602-2 (accession # AM293860) using MAQ and the same criteria as above.

### Bioinformatic alnalysis

Functional analysis and annotations analysis of the *V. vulnificus *YJ016 and CMCP6 genes were done using the Pathway Tool Omics viewer from the BioCyc platform [81] and the SEED database [82].

## Authors' contributions

PAG planned and coordinated the research, analyzed data, and wrote the manuscript. VDC contributed to data analysis and writing. ACW contributed to planning and writing. BW performed MAQ data analysis and planning. MTS contributed to the writing the manuscript. LMM helped plan the study, planned analyses, and contributed to the writing of the manuscript. All authors read and approved the final manuscript.

## Supplementary Material

Additional file 1 **Table S1: Coverage of the *V. vulnificus *biotype 2 plasmids by newly sequenced reads**. SOLiD sequencing reads of each of the four newly sequenced genomes were matched with the three plasmids of *V. vulnificus *biotype 2 using MAQ. The size of each plasmid is shown. *Numbers of nucleotides of the reference plasmid with less than 10-fold coverage by 35-nt reads from the newly sequenced genome. **Number of nucleotides that were matched by virtue of having 10-fold or greater coverage depth. ***Percent of reference plasmid matched to the newly sequenced genome. Click here for file

Additional file 2 **Table S2: Identification of ORFs in newly sequenced *V. vulnificus *genomes by matching with the *V. cholerae *NC16961 genome**. SOLiD sequencing reads of each of the four newly sequenced genomes were matched with the *V. cholerae *NC16961 using MAQ, as described in the Methods. *V. vulnificus *strains: M06 - M06-24/O, B5 - 99-738 DP-B5, B8 - 99-520 DP-B8, ATCC - ATCC 33149. Genes were considered matched if there was five-fold or higher depth of coverage over five tandem 100-nt windows. Click here for file

Additional file 3 **Table S3A: Matches of CMCP6 genes from the YJ016 reference genome and the four newly sequenced genomes**. The CMCP6 genes are shown by their tag, gene name (if annotated), and product (if known). Matching of each gene with the newly sequenced genomes was determined using MAQ, as described in the Methods. Matches with the YJ016 genome were obtained using GenPlot at http://www.ncbi.nlm.nih.gov using default parameters. Genes from each queried genome that were not matched to the CMCP6 genome are indicated with an X. If a CMCP6 gene is missing from all of the other five genomes, it is indicated with an × in the CMCP6-Specific column. *V. vulnificus *strains: M06 - M06-24/O, B5 - 99-738 DP-B5, B8 - 99-520 DP-B8, ATCC - ATCC 33149. Click here for file

Additional file 4 **Table S3B: Matches of YJ016 genes from the CMCP6 reference genome and the four newly sequenced genomes**. The YJ016 genes are shown by their tag, gene name (if annotated), and product (if known). Matching of each gene with the newly sequenced genomes was determined using MAQ, as described in the Methods. Matches with the CMCP6 genome were obtained using GenPlot at http://www.ncbi.nlm.nih.gov using default parameters. Genes from each queried genome that were not matched to the YJ016 genome are indicated with an X. If a YJ016 gene is missing from all of the other five genomes, it is indicated with an × in the YJ016-Specific column. *V. vulnificus *strains: M06 - M06-24/O, B5 - 99-738 DP-B5, B8 - 99-520 DP-B8, ATCC - ATCC 33149. Click here for file

Additional file 5 **Table S4: The core *V. vulnificus *genome**. Genes that were present in the two reference genomes and each of the four newly sequenced genomes are shown using the CMCP6 tag, product, gene name, and cog. Click here for file

Additional file 6 **Table S5A: SNP analysis of *V. vulnificus *M06-24/O compared with the CMCP6 reference genomes**. MAQ was used to identify SNPs from the SOLiD sequencing reads from M06-24/O compared with the CMCP6 reference genome, as described in the Methods. Pos. - Position of the nucleotide in the genomic element. Ref. - Reference base in the reference genome. Con. - Consensus base in the newly sequenced genome. Con. QS - Consensus Quality Score. Read depth - Depth of coverage at the chosen nucleotide. Avg. hits - Average number of hits of reads covering the position. HMQ - Highest mapping quality of reads covering the position. MCQ - Minimum consensus quality in the third flanking region on each side of the site. 2nd - second best call for the nucleotide. LLR - Log likelihood ratio of the second and third best call. 3rd - Third best call. Click here for file

Additional file 7 **Table S5B: SNP analysis of *V. vulnificus *M06-24/O compared with the YJ016 reference genome**. MAQ was used to identify SNPs from the SOLiD sequencing reads from M06-24/O compared with the YJ016 reference genome, as described in the Methods. Column headings are as for Additional File 6, Table S5A. Click here for file

Additional file 8 **Table S6A: SNP analysis of *V. vulnificus *99-738 DP-B5 compared with the CMCP6 reference genome**. MAQ was used to identify SNPs from the SOLiD sequencing reads from 99-738 DP-B5 compared with the CMCP6 reference genome, as described in the Methods. Column headings are as for Additional File 6, Table S5A. Click here for file

Additional file 9 **Table S6B: SNP analysis of *V. vulnificus *99-738 DP-B5 compared with the YJ016 reference genome**. MAQ was used to identify SNPs from the SOLiD sequencing reads from 99-738 DP-B5 compared with the YJ016 reference genome, as described in the Methods. Column headings are as for Additional File 6, Table S5A. Click here for file

Additional file 10 **Table S7A: SNP analysis of *V. vulnificus *99-520 DP-B8 compared with the CMCP6 reference genome**. MAQ was used to identify SNPs from the SOLiD sequencing reads from 99-520 DP-B8 compared with the CMCP6 reference genome, as described in the Methods. Column headings are as for Additional File 6, Table S5A. Click here for file

Additional file 11 **Table 7B: SNP analysis of *V. vulnificus *99-520 DP-B8 compared with the YJ016 reference genome**. MAQ was used to identify SNPs from the SOLiD sequencing reads from 99-520 DP-B8 compared with the YJ016 reference genome, as described in the Methods. Column headings are as for Additional File 6, Table S5A. Click here for file

Additional file 12 **Table 8A: SNP analysis of *V. vulnificus *ATCC 33149 compared with the CMCP6 reference genome**. MAQ was used to identify SNPs from the SOLiD sequencing reads from ATCC 33149 compared with the CMCP6 reference genome, as described in the Methods. Column headings are as for Additional File 6, Table S5A. Click here for file

Additional file 13 **Table 8B: SNP analysis of *V. vulnificus *ATCC 33149 compared with the YJ016 reference genome**. MAQ was used to identify SNPs from the SOLiD sequencing reads from ATCC 33149 compared with the YJ016 reference genome, as described in the Methods. Column headings are as for Additional File 6, Table S5A. Click here for file
