# Supplementary material for: SOLiD sequencing of four Vibrio vulnificus genomes enables comparative genomic analysis and identification of candidate clade-specific virulence genes
Source: BMC Genomics. 2010 Sep 24;11:512. doi: 10.1186/1471-2164-11-512 (PMC3091676; doi:10.1186/1471-2164-11-512)
Supplement: Additional file 1 — Table S1: Coverage of the V. vulnificus biotype 2 plasmids by newly sequenced reads. SOLiD sequencing reads of each of the four newly sequenced genomes were matched with the three plasmids of V. vulnificus biotype 2 using MAQ. The size of each plasmid is shown. *Numbers of nucleotides of the reference plasmid with less than 10-fold coverage by 35-nt reads from the newly sequenced genome. **Number of nucleotides that were matched by virtue of having 10-fold or greater coverage depth. ***Percent of reference plasmid matched to the newly sequenced genome. [file 1471-2164-11-512-S1.PDF]

Additional Table 1. Coverage of the *V. vulnificus* biotype 2 plasmids by newly sequenced reads.

		ATCC33149			99-738 DP-B5			99-520 DP-B8			M06-24/O		
**Plasmid**	**size (bp)**	**<10***	**matched****	**%*****	**<10**	**matched**	**%**	**<10**	**matched**	**%**	**<10**	**matched**	**%**
**pC4602-1**	56,628	4,384	52,244	92	24,807	31,821	56	53,219	3,409	6	56,628	0	0
**pC4602-2**	66 946	4 005	62 941	94	40 310	26 636	40	54 114	12 832	19	66 229	717	1
