# Supplementary material for: SOLiD sequencing of four Vibrio vulnificus genomes enables comparative genomic analysis and identification of candidate clade-specific virulence genes
Source: BMC Genomics. 2010 Sep 24;11:512. doi: 10.1186/1471-2164-11-512 (PMC3091676; doi:10.1186/1471-2164-11-512)
Supplement: Additional file 2 — Table S2: Identification of ORFs in newly sequenced V. vulnificus genomes by matching with the V. cholerae NC16961 genome. SOLiD sequencing reads of each of the four newly sequenced genomes were matched with the V. cholerae NC16961 using MAQ, as described in the Methods. V. vulnificus strains: M06 - M06-24/O, B5 - 99-738 DP-B5, B8 - 99-520 DP-B8, ATCC - ATCC 33149. Genes were considered matched if there was five-fold or higher depth of coverage over five tandem 100-nt windows. [file 1471-2164-11-512-S2.PDF]

**Additional Table 2. Identification of ORFs in newly sequenced *V. vulnificus* genomes by matching with the *V. cholerae* NC16961 genome.**

| *V. vulnificus* Strain | | | | *V. cholerae* NC16961 genome match | | |
|---|---|---|---|---|---|---|
| ATCC | B5 | B8 | M06 | tag | gene | product |
| X | | X | | VC0255 | - | rfbT-related protein |
| | | X | | VC0256 | - | transposase OrfAB, subunit A |
| X | | | | VC0496 | - | hypothetical protein VC0496 |
| X | | | | VC0497 | - | transcriptional regulator |
| X | | | | VC0504 | - | hypothetical protein VC0504 |
| X | | | | VC0505 | - | hypothetical protein VC0505 |
| X | | | | VC0507 | - | hypothetical protein VC0507 |
| X | | | | VC0508 | - | hypothetical protein VC0508 |
| X | | | | VC0509 | - | hypothetical protein VC0509 |
| X | | | | VC0516 | - | phage integrase |
| | | X | | VC1477 | - | transposase OrfAB, subunit A |
| | | X | | VC1790 | - | transposase OrfAB, subunit A |
| | | X | | VC1791 | - | hypothetical protein VC1791 |
| X | | | | VCA0294 | - | hypothetical protein VCA0294 |
| | X | | | VCA0301 | - | short chain dehydrogenase/reductase family oxidoreductase |
| X | | X | | VCA0332 | - | hypothetical protein VCA0332 |
| X | | X | | VCA0333 | - | hypothetical protein VCA0333 |
| X | | X | | VCA0334 | - | hypothetical protein VCA0334 |
| | | X | | VCA0372 | - | transposase OrfAB, subunit A |
| | | X | | VCA0373 | - | hypothetical protein VCA0373 |
| X | | | | VCA0391 | - | killer protein, putative |
| X | | | | VCA0392 | - | antidote protein, putative |
| | | | X | VCA0431 | - | hypothetical protein VCA0431 |
| | | | X | VCA0432 | - | hypothetical protein VCA0432 |
| | | | X | VCA0433 | - | hypothetical protein VCA0433 |
| | | X | | VCA0443 | - | lipoprotein Blc |
| | | X | | VCA0474 | - | acetyltransferase, putative |
| | | X | | VCA0507 | - | transposase OrfAB, subunit A |
| | | X | | VCA0792 | - | transposase OrfAB, subunit A |
| | | X | | VCA0793 | - | hypothetical protein VCA0793 |
